# Defying the Prognostic Odds: A Case Report of Unexpected Complete Remission of Metastatic Ampullary Carcinoma With Palliative Chemotherapy

**DOI:** 10.1155/crom/7884410

**Published:** 2025-09-09

**Authors:** Akil Olliverrie, Joel Gabin Konlack Mekontso, Jingwei Ren, Syed Karim, Imad Karam, Edwin Chiu, Christopher Chum

**Affiliations:** ^1^Department of Internal Medicine, NYC Health and Hospitals South Brooklyn Health, Brooklyn, New York, USA; ^2^OMS-4, Touro University College of Osteopathic Medicine, Harlem, New York, USA; ^3^Department of Gastroenterology, NYU Langone Health, Brooklyn, New York, USA; ^4^Department of Hematology and Oncology, NYC Health and Hospitals Kings County Hospital, Brooklyn, New York, USA; ^5^Department of Gastroenterology, NYC Health and Hospitals South Brooklyn Health, Brooklyn, New York, USA

**Keywords:** complete cancer remission, metastatic ampullary carcinoma, PALB2 mutation, palliative chemotherapy, prognosis

## Abstract

Metastatic ampullary carcinoma (AC) almost always carries a poor prognosis. We present a remarkable case of a 69-year-old male with Stage IV pancreaticobiliary-type AC who achieved a complete remission after 45 months of palliative modified FOLFIRINOX chemotherapy (5-fluorouracil, oxaliplatin, leucovorin, irinotecan). This unexpected outcome challenges the conventional understanding of the natural history of advanced AC. Furthermore, molecular analysis revealed a pathogenic PALB2 mutation, along with variants of unknown significance in the POLD1 and RAD50 genes, coding for enzymes involved in various deoxyribonucleic acid (DNA) repair pathways. These findings raise questions about their potential influence on treatment response and prognosis. This case underscores the need for further investigation into the role of molecular alterations and personalized approaches in managing advanced AC.

## 1. Introduction

Ampullary carcinoma (AC), a rare malignancy accounting for only 6% of periampullary cancers, often presents a therapeutic challenge, especially in its metastatic form [1]. While surgery offers the best chance for cure in localized disease [2], the prognosis for Stage IV AC remains dismal, with 5-year survival rates approaching zero [3]. We report an exceptional case of a patient with Stage IV pancreaticobiliary-type AC who achieved a durable complete remission exceeding 4 years with palliative chemotherapy alone—a highly unusual outcome. This case highlights the potential influence of molecular alterations, specifically a pathogenic PALB2 mutation, along with variants in the POLD1 and RAD50 genes, on treatment response and prognosis. This report adheres to the Case Report (CARE) guidelines [4].

## 2. Case Description

We present a 69-year-old male with hypertension and hyperlipidemia who was referred to the emergency room by his primary care physician (PCP) in December 2019 for evaluation of abnormal liver function tests. Since mid-November, he had experienced intermittent abdominal pain, decreased appetite, significant fatigue with minimal activity, progressing to pale stools, dark urine, painless jaundice, and reduced exercise capacity.

He last saw his PCP 6 months ago. During that visit, his physical examination and laboratory workup, including a complete blood count and comprehensive chemistry panel, were unremarkable. He reported consuming alcohol once a month and denied smoking, recent travel, or contact with dogs, cats, or cattle. Family history was significant for liver cancer in a nephew but revealed no relevant genetic conditions. His medications included daily amlodipine 10 mg and atorvastatin 40 mg. He also denied using any over-the-counter medications or herbal remedies.

On physical examination, the blood pressure was 150/88 mmHg, with a stable weight of 205 lbs. All other vital signs, including the body mass index, were within normal limits. Scleral and sublingual icterus were observed. The abdomen was moderately distended, with mild generalized tenderness to palpation, without rebound or guarding. Laboratory investigations revealed abnormal liver function tests: alanine aminotransferase 411 U/L, aspartate aminotransferase 687 U/L, alkaline phosphatase 543 U/L, total bilirubin 26.9 mg/dL, direct bilirubin 18.1 mg/dL, and albumin 3.1 g/dL. The differential diagnosis included neoplastic, infectious, and inflammatory processes.

**Figure 1:**
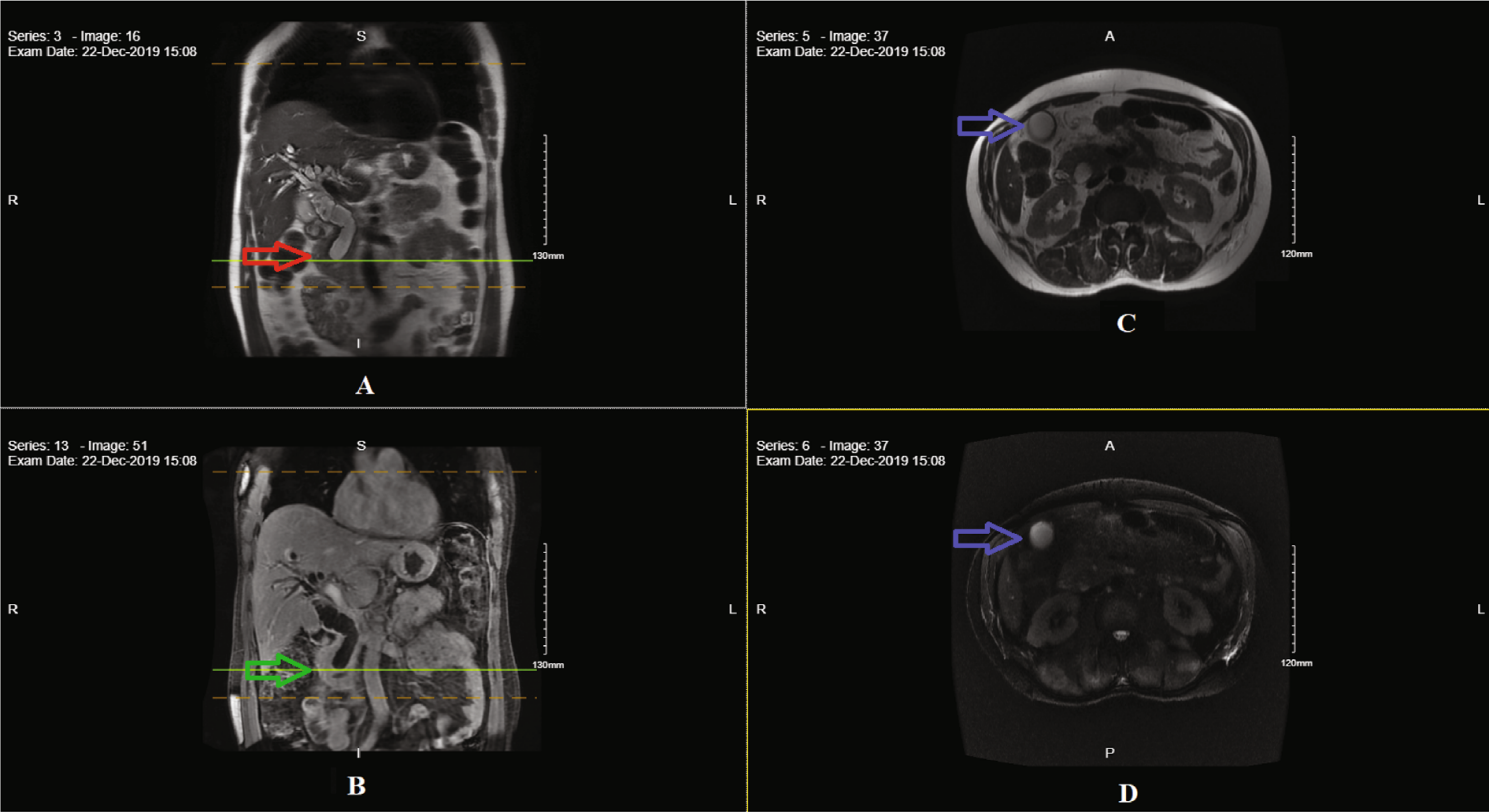
MRCP images (coronal and transverse sections) demonstrate intrahepatic and extrahepatic biliary ductal dilatation with a mass lesion at the ampulla of Vater (red and green arrows). Additionally, a nonspecific nodule is observed in the right liver lobe (blue arrows).

Imaging studies conducted the following day, including a computed tomography (CT) scan of the abdomen and pelvis and a magnetic resonance cholangiopancreatography (MRCP), revealed an ampullary mass measuring 1.9 × 1.6 × 2.9 cm. This was associated with severe dilation of the intrahepatic and extrahepatic bile ducts, with abrupt tapering at the ampulla of Vater (Figure 1a,b). Additionally, a 1.8 × 1.2 cm nodule was detected in the right liver lobe (Figure 1c,d). No enlarged retroperitoneal, mesenteric, or pelvic lymph nodes were observed.

**Figure 2:**
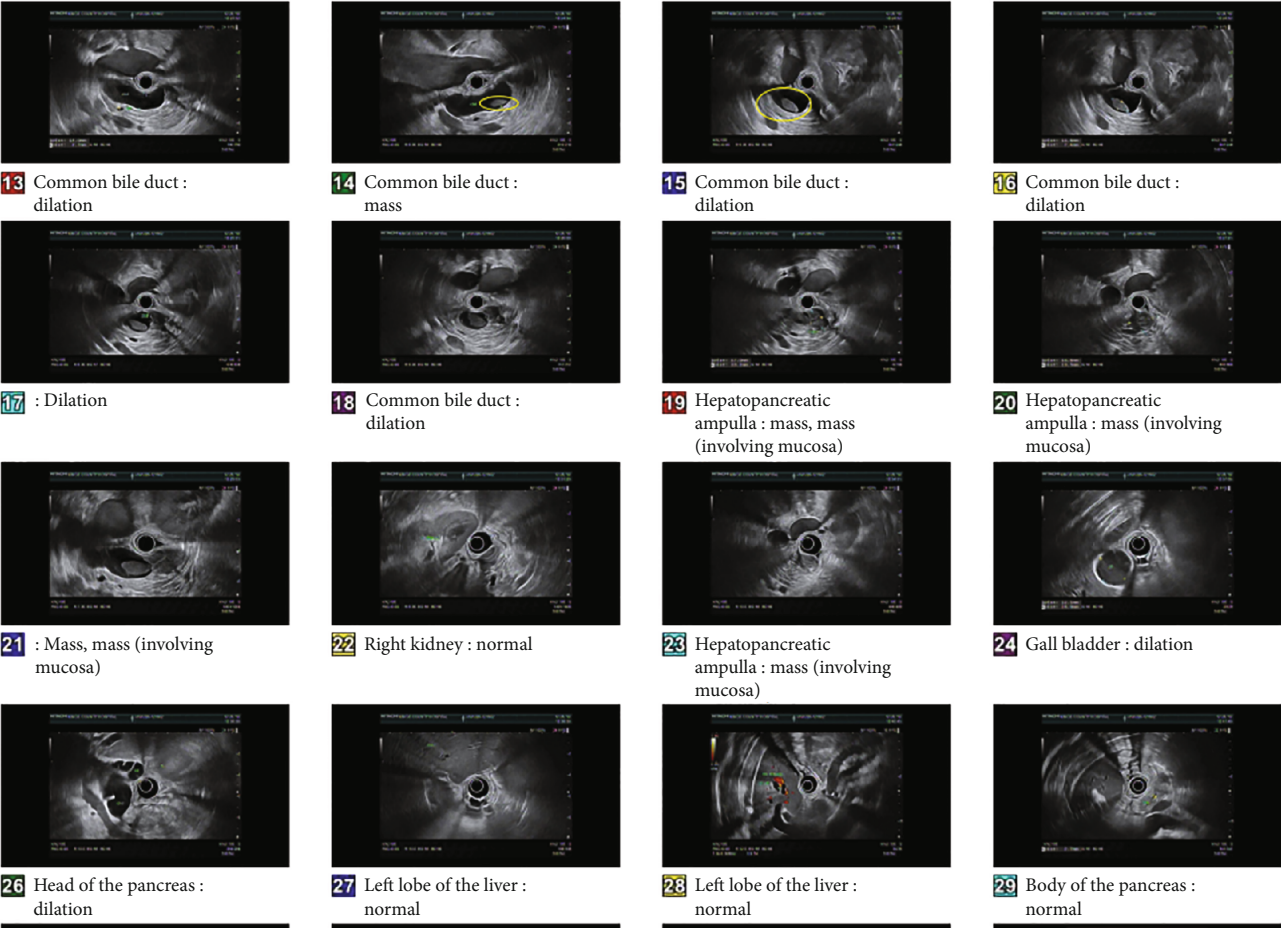
EUS showing dilation of the CBD and a mucosal ampullary mass.

Endoscopic ultrasonography (EUS) on Hospital Day 5 revealed an ampullary mass extending into the common bile duct (CBD) as depicted in Figure 2. A sphincterotomy with CBD stenting was performed, and brush biopsies confirmed the presence of malignant cells. Immunohistochemical analysis of a CT-guided liver biopsy identified tumor cells positive for cytokeratin (CK) 7, CK17, and CK19, consistent with adenocarcinoma of pancreaticobiliary origin. A Whipple procedure was deemed unsuitable due to the presence of a liver lesion, which also tested positive for adenocarcinoma. The diagnosis of metastatic AC was thus established, with a poor prognosis. Molecular profiling was performed to further characterize the tumor and identify potential therapeutic targets. The patient underwent germline genetic testing via the Cancer Health Equity Research Program at Memorial Sloan Kettering Cancer Center. This testing, conducted by BE-Genetics in May 2020, identified a heterozygous pathogenic mutation in the **PALB2** gene [p.S524∗]. The analysis also revealed two variants of unknown significance in the **POLD1** [c.-5G>C] and **RAD50** [p.R87C] genes. The tumor was negative for mutations in poly ADP-ribose polymerase (PARP) and mismatch repair (MMR) genes, and no significant genetic alterations were identified in the programmed death-1 (PD-1) and programmed death-ligand 1 (PDL-1) genes.

His case was discussed in a multidisciplinary tumor board in January 2020, where the consensus was to initiate palliative chemotherapy without radiation therapy. The patient reported manageable pain and discomfort, with no acute distress observed. He underwent palliative chemotherapy with a modified FOLFIRINOX regimen (5-fluorouracil [5-FU], oxaliplatin, leucovorin, irinotecan), tailored to his pancreaticobiliary-type histology, from March 2020 to December 2023. The original regimen consisted of oxaliplatin at 85 mg/m^2^ of body surface area, irinotecan at 180 mg/m^2^, leucovorin at 400 mg/m^2^, and fluorouracil at 1200 mg/m^2^ administered every 3 weeks. However, due to dose-limiting neutropenia and thrombocytopenia, the oxaliplatin dose was reduced to 75 mg/m^2^ starting at Cycle 8 and further reduced to 60 mg/m^2^ beginning at Cycle 15. The irinotecan dose was also reduced to 120 mg/m^2^ from Cycle 15 onwards. At the patient's request due to ongoing side effects, the treatment interval was extended to every 4 weeks starting with Cycle 31. Finally, from Cycle 39 onwards, irinotecan was discontinued, and the patient was maintained on a FOLFOX regimen (5-FU, oxaliplatin, and leucovorin). His treatment included scheduled drug holidays from December 21, 2020, to January 25, 2021; July 7, 2021, to August 17, 2021; and December 21, 2021, to February 1, 2022. He completed 2 out of 12 cycles with the original dose and 47 out of 50 cycles with a reduced dose. Chemotherapy was continued based on radiologic improvement until the patient requested discontinuation due to persisting and unbearable side effects.

**Figure 3:**
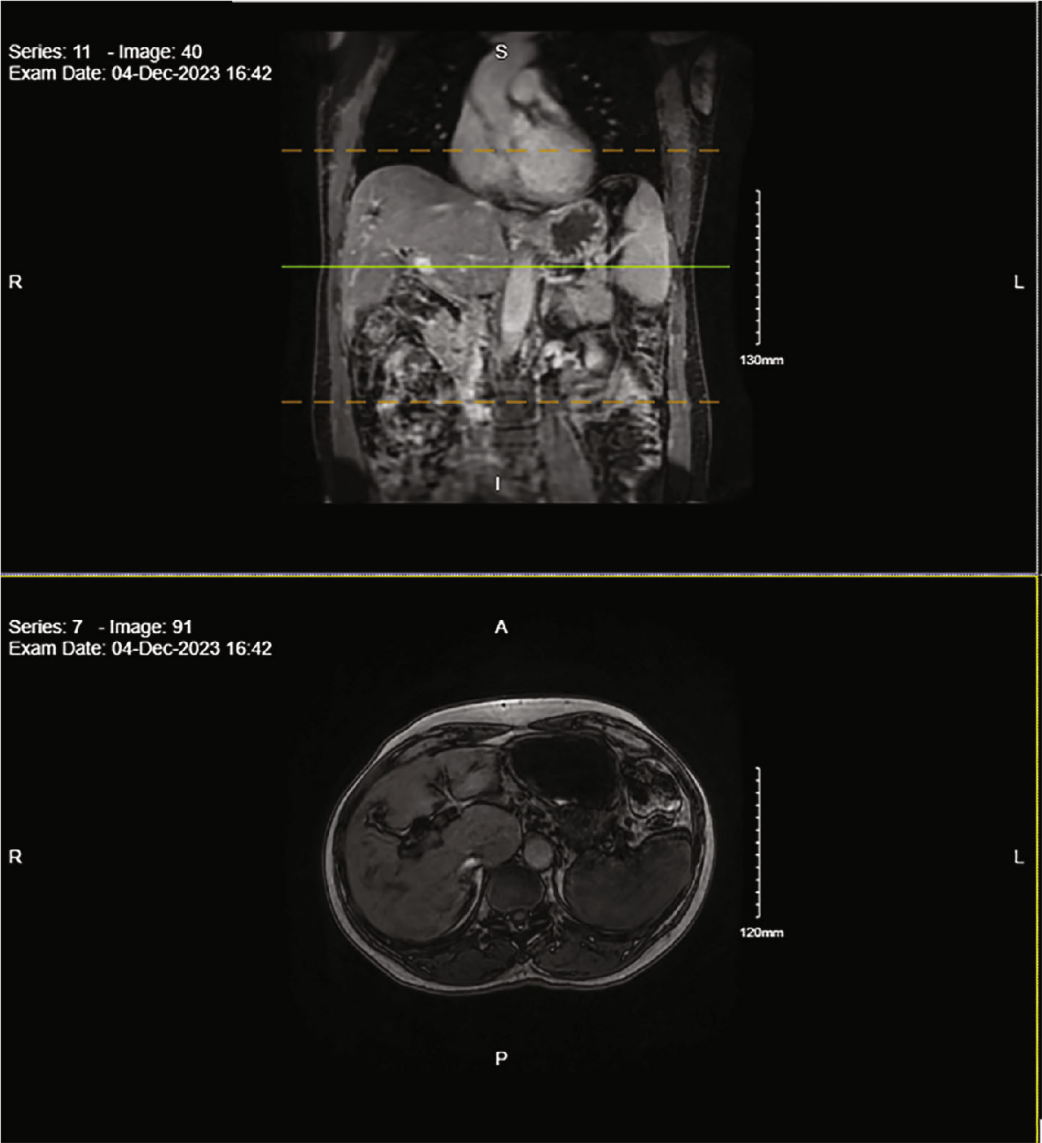
MRCP demonstrating mild intrahepatic ductal dilatation with no appreciable enhancing lesions in the liver or biliary system.

**Figure 4:**
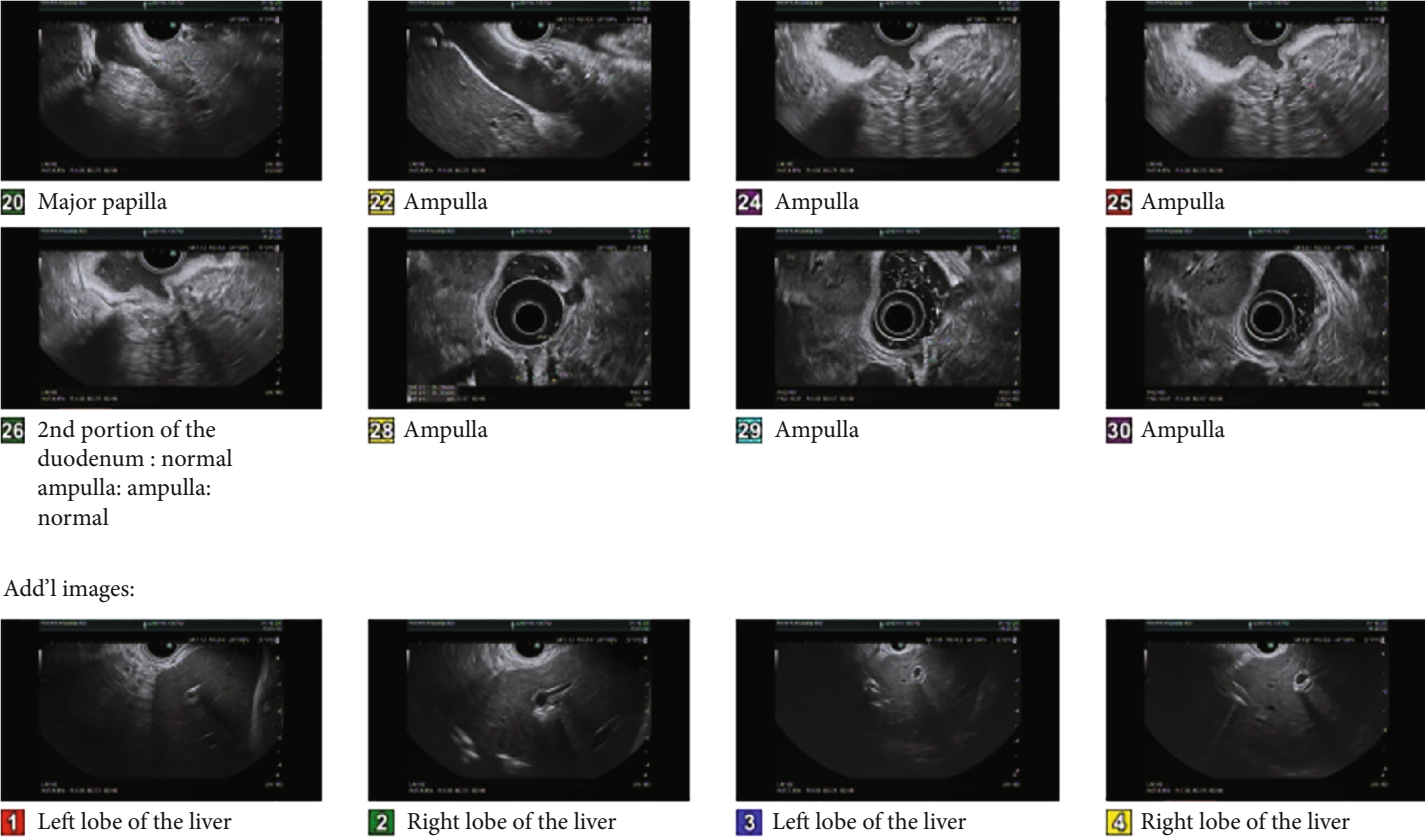
EUS showing no significant endosonographic abnormalities in the ampulla. A prior sphincterotomy site was noted. No pathological lymphadenopathy, masses, or wall thickening was identified.

Surveillance MRCP conducted in December 2023 (Figure 3) and EUS in January 2024 (Figure 4) postchemotherapy revealed no detectable liver, ampullary, or biliary system lesions. An ERCP performed the same day confirmed the absence of lesions, with biopsies showing no evidence of dysplasia or malignancy. He has since maintained regular oncology follow-ups. He remained asymptomatic as of his last visit in October 2024. A mammogram performed in March 2024 was categorized as BIRADS-2, with the next one scheduled for March 2025.

## 3. Discussion

AC originates at the ampulla of Vater, positioned distally to the junction between the pancreatic duct and the CBD. This gastrointestinal malignancy is rare, showing a slightly higher incidence in males and presenting across a wide age spectrum at diagnosis. It accounts for only 0.2% of gastrointestinal cancers and contributes to 7%–20% of all periampullary cancers [5, 6]. Histologically, there are three main subtypes: intestinal, pancreaticobiliary, and mixed, each characterized by unique features and prognostic implications [6]. Pancreaticobiliary AC is the most prevalent subtype, constituting 50%–60% of cases, yet it carries a poorer prognosis compared to the intestinal subtype [7]. A retrospective study has identified advanced stage and CK7+/CK20− expression as independent factors associated with lower overall survival rates [8]. In our case, the patient presents with Stage IV disease and CK7+/CK20−/CK17+/CK19+ expression, signifying an advanced and concerning scenario regarding ampullary cancer.

Surgical resection via the Whipple procedure is a prevalent treatment modality for initial-stage AC, although postoperative tumor recurrence occurs in roughly 50% of cases. Chemotherapy is conventionally employed in the management of AC, particularly in instances involving distant metastases, recurrence, or inoperable local advancement [9–11]. Treatment efficacy, however, remains suboptimal for patients with advanced ampullary adenocarcinomas. A retrospective cohort study reported on the administration of chemotherapeutic agents to 12 patients with advanced-stage and 14 with recurrent ampullary cancer, utilizing regimens based on either 5-FU or gemcitabine. The study found a median overall survival of 8.0 months in the 5-FU cohort and 12.3 months in the gemcitabine cohort [9]. An alternative chemotherapeutic protocol, the XELOX regimen (combining oxaliplatin with capecitabine), was investigated in a separate study where 21 patients with recurrent or metastatic AC were treated and monitored over an average of 16.6 months. Overall, the median time to progression was 7.6 months (95% confidence interval [CI], 6.7–8.5). However, it was noted that the median time to progression for patients with the pancreaticobiliary variant was significantly shorter than for those with intestinal-type adenocarcinoma (6.4 vs. 13.1 months, *p* = 0.038) [11].

A case study from 2014 outlined a scenario closely comparable to ours, involving the use of the same chemotherapy drug for a patient with a similar stage of ampullary adenocarcinoma. The report documented a promising response to 5-FU treatment in a patient diagnosed with advanced-stage T4N0M1 ampullary adenocarcinoma, highlighting the absence of radiological disease evidence 12 months postdiagnosis. It was noted that chemotherapy was administered after the patient underwent a Whipple pancreatoduodenectomy [10]. In contrast, our present case involves a patient with a similar stage who received 5-FU-based therapy without prior surgery. Remarkably, he demonstrated a rare and prolonged period of disease control lasting more than 4 years. This difference in treatment approach (chemotherapy alone vs. chemotherapy after surgery) and the exceptionally long survival in our patient highlight the unusual nature of this case.

Certain factors play an important role in the poor prognosis of AC. There is an increasing focus on leveraging the molecular profile of AC to refine prognostic assessments and identify potential therapeutic targets. Notably, mutations in genes such as P53 and K-RAS are established as adverse prognostic markers [12]. However, our patient does not exhibit indications of P53 and K-RAS mutations but instead harbors a PALB2 mutation, along with variants of the POLD1 and RAD50 genes. While mutations in the PALB2 gene have been associated with an increased risk of breast cancer in males [13], its role in other cancers, including AC, remains poorly understood. The POLD1 gene codes for a subunit of the deoxyribonucleic acid (DNA) polymerase delta complex, which is vital for DNA replication, particularly in synthesizing the lagging strand. This complex also participates in various DNA repair pathways, including base excision, double-strand break, and MMR [14]. Similarly, the protein coded by the RAD50 gene is involved in DNA double-strand break repair [15]. This case raises the hypothesis that these genetic alterations may have contributed to the patient's unusually positive response to chemotherapy. Further research is needed to investigate the potential link between PALB2, POLD1, and RAD50 mutations or alterations and chemosensitivity in AC. Beyond these molecular markers, other established prognostic factors such as moderate or poor tumor differentiation, tumor size exceeding 2 cm, and the presence of lymph node metastasis are frequently linked to a poorer prognosis [16]. In our case, the patient demonstrates an absence of local adenopathy, and the tumor size was slightly above the adverse prognostic threshold.

The selection of chemotherapy for AC poses a challenge due to the limited availability of comprehensive data, primarily owing to the rarity of this malignancy. Existing chemotherapy data, especially for advanced cases, are often amalgamated with data from patients diagnosed with small bowel, pancreatic, and ampullary adenocarcinomas or, more commonly, AC combined with biliary tract cancers. However, given the recent advancements in the management of advanced disease, accurate differentiation between periampullary tumors originating from the intestinal, biliary, or pancreatic regions is critical for tailoring treatment strategies effectively. Despite ongoing efforts, a consensus regarding the optimal management of metastatic disease in genuine ACs has yet to be established. Nonetheless, it is recommended that all potential candidates for systemic targeted therapy undergo next-generation sequencing of tumor tissue and receive referrals for germline genomic testing. This approach is essential as it enables the identification of molecular alterations that could inform the selection of patients who could benefit from molecularly targeted therapy [17].

## 4. Conclusion

This case challenges the traditionally poor prognosis of metastatic AC and raises important questions regarding the role of molecular alterations in determining treatment response. While no current evidence directly supports it, we hypothesize that the presence of a PALB2 mutation, along with variants in the POLD1 and RAD50 genes, may have played a role in the patient's exceptional outcome, highlighting the need for further studies on the prognostic and predictive value of such genetic markers. Additionally, this case underscores the importance of considering individualized chemotherapy strategies, as even aggressive histologic subtypes may exhibit unexpected responses. Further research is warranted to refine prognostic models and optimize treatment approaches for metastatic AC.

## Data Availability

The data that support the findings of this study are available on request from the corresponding author. The data are not publicly available due to privacy or ethical restrictions.
